# Biobank Alzheimer Center Limburg study: A multimodal longitudinal dementia biobank

**DOI:** 10.1177/25424823261496546

**Published:** 2026-10-08

**Authors:** Inez H.G.B. Ramakers, Leonie C.P. Banning, Willemijn J. Jansen, Lieke Bakker, Ron L.H. Handels, Sebastian Köhler, Pieter Jelle Visser, Stephanie J.B. Vos, Pauline Aalten, Frans R.J. Verhey

**Affiliations:** 1 Department of Psychiatry and Neuropsychology, School for Mental Health and Neuroscience, Alzheimer Center Limburg, 82246 Maastricht University, Maastricht, the Netherlands

**Keywords:** Alzheimer's disease, biomarkers, cohort study, dementia, diagnosis, memory clinic, mild cognitive impairment, prognosis, subjective cognitive decline

## Abstract

**Background:**

There is an urgent need to better understand the mechanisms of dementia, particularly Alzheimer's disease (AD), the most common subtype. The BioBank Alzheimer Center Limburg (BBACL) study is an ongoing prospective cohort, including consecutive patients from the memory clinic of the Alzheimer Center Limburg, Maastricht University Medical Center+, the Netherlands. It aims to investigate the natural course of cognitive complaints and identify early clinical and biological determinants of disease progression.

**Objective:**

To describe the BBACL study design and procedures and present the baseline characteristics of the currently included participants.

**Methods:**

Patients undergo standardized assessment, including demographics, comorbidities, cognitive and functional assessments, neuropsychiatric symptoms, MRI cerebrum, and biomaterials [blood and cerebrospinal fluid (CSF) samples (subset)]. Additional blood and CSF samples are stored for future biomarkers research. Follow-up is scheduled at 1, 2, 5, and 10 years.

**Results:**

Since its start in 2009, 920 patients have been included at baseline. The mean baseline age was 68.4 (SD 11.3) years, with 41.6% being females. The mean Mini-Mental State Examination score was 27.0 (SD 2.6). At baseline, 39.6% had subjective cognitive decline, 40.0% mild cognitive impairment, and 20.4% dementia (58.0% probable/possible AD and 19.7% mixed AD/vascular dementia), reflecting the study's emphasis on early diagnostics. Cognitive, functional, and biomarker measures varied across diagnostic groups as expected.

**Conclusions:**

BBACL combines extensive biospecimens with rich multimodal phenotyping and longitudinal follow-up, creating opportunities for future integrative analyses and omics-based analyses to uncover novel biological signatures.

## Introduction

Dementia is one of the largest health care challenges in the Western world, affecting more than 55 million people worldwide in 2021.^1^ The disease has considerable impact on individuals, their caregivers (e.g., quality of life and mental health) and society, with estimated worldwide total costs of US$604 billion.^2^ The clinical syndrome is characterized by cognitive decline and behavioral problems, progressively interfering with activities in daily life.^3^

It is now known that Alzheimer's disease (AD), the most common cause of dementia, has a disease onset that starts decades before clinical symptoms emerge.^4^ The transition phase between normal aging and dementia has been described by the concepts of subjective cognitive decline (SCD) and mild cognitive impairment (MCI).^5,6^ However, not all individuals with SCD or MCI develop dementia, and those who do develop dementia show a variety in onset.^7^ A multifactorial approach including biological and psychosocial aspects is needed to explain this heterogeneity in the prognosis of people with cognitive complaints or impairments. It is important to increase our understanding of factors that are associated with etiology and prognostic outcomes, as this improves an early diagnosis of neurodegenerative disorders, which creates a time-window to provide timely interventions and facilitates individualized care for patient and caregivers.^8^

The BioBank Alzheimer Center Limburg (BBACL) study encompasses longitudinal data from patients from the Maastricht memory clinic across the clinical disease spectrum ranging from SCD to mild dementia, and is integrated in regular patient care. BBACL measures a wide range of clinical and biological parameters at baseline and has a long clinical follow-up period of up to ten years, which enables identifying markers for early diagnostics and prognostics of AD dementia. Here we provide a detailed outline of the BBACL study design and protocol, and an overview of the baseline characteristics of the currently included patients.

## Methods

### Objectives

The objectives of BBACL are threefold: (1) to describe the natural course of cognitive functioning, neuropsychiatric symptoms, daily functioning, and diagnostic outcomes, such as the development of MCI and dementia in memory clinic visitors; (2) to identify determinants for the course and outcomes, such as clinical parameters and existing and newly identified biomarkers of pathology; and (3) to identify factors associated to health economic outcomes such as care use and quality of life.

### Study design and setting

The BBACL cohort is an ongoing, prospective clinical cohort of patients referred to the memory clinic of the Maastricht University Medical Center (MUMC+), the Netherlands and is hosted by the Alzheimer Center Limburg (ACL). The ACL is a top-referent, academic center in the south of the Netherlands, integrating clinical (MUMC+) and research activities (Maastricht University) in the field of cognitive disorders, including dementia. More specifically, ACL has three research lines including: early diagnosis and disease mechanisms, risk and prevention, and psychosocial innovations.

The BBACL cohort includes patients who are referred by the general practitioner to the memory clinic with signs of cognitive deficits ranging from SCD to (mild) dementia. The BBACL cohort was initiated in 2009 and recruitment is currently ongoing, with all consecutive patients being evaluated for study eligibility at the time of their first visit. Patients then are invited to the one-day diagnostic program of the memory clinic (i.e., a baseline assessment), which is guided by a research nurse and includes extensive clinical, neurological, neuropsychological, neuropsychiatric, and blood assessments, and an MRI cerebrum (Figure 1). Additional data is collected from the caregiver. Follow-up assessments are planned after one, two, five and ten years.

**Figure 1.**
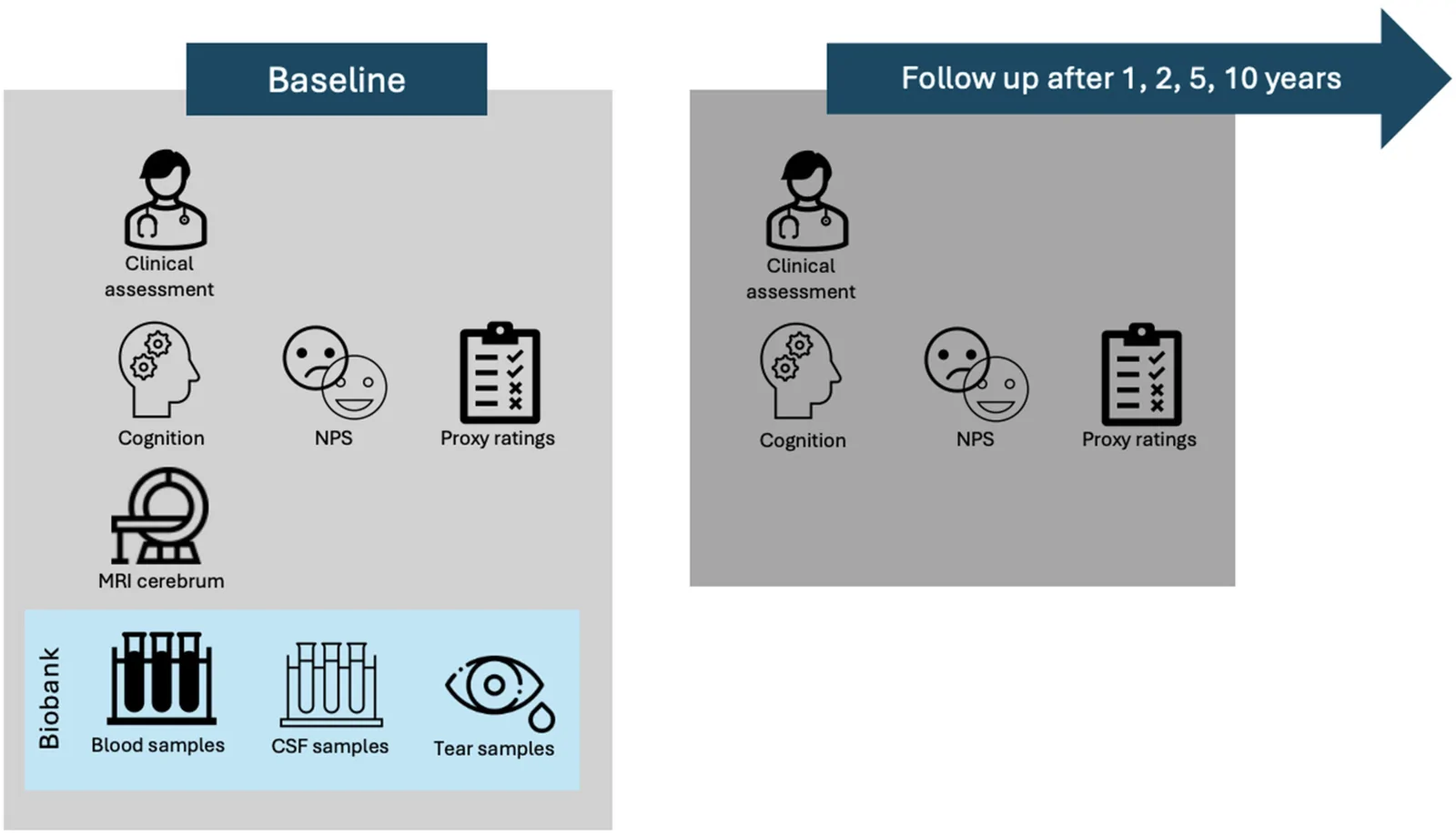
Overview of assessments of the BBACL cohort.

### Eligibility criteria

Inclusion criteria are a Clinical Dementia Rating scale (CDR)^9^ score from 0 to 1, and a Mini-Mental State Examination (MMSE)^10^ score ≥20, thereby including patients across the clinical spectrum of SCD, MCI and mild dementia. Exclusion criteria at baseline are non-degenerative neurological diseases, such as normal pressure hydrocephalus, Morbus Huntington, brain tumor, epilepsy, encephalitis, recent transient ischemic attack (TIA) or cerebrovascular accident (CVA) (<2 years), or TIA/CVA with concurrent (within three months) cognitive decline; a history of a severe mental disorder such as schizophrenia, bipolar disorder or psychotic problems (not otherwise specified), current major depressive disorder (within 12 months), or alcohol abuse; and the expectation that a follow-up assessment after one year will not be possible (e.g., due to a fatal disease). Participants who are diagnosed with dementia are not invited for further follow-up. Written informed consent is collected from all participants to store and use pseudonymized clinical data and biomaterials (blood and cerebrospinal fluid (CSF)). Data storage takes place in a secure electronic record system that facilitates data sharing for re-use. Biomaterials are stores in the MUMC + ISO-certified biobank with the aim to answer future research questions.

### Baseline assessment

At baseline, all patients undergo a standardized assessment according to predefined Standard Operating Procedures (SOP), elaborated upon below and shown in Supplemental Table 1.

*Demographics.* Demographic variables include age, sex, ethno-racial status, years of education, and living situation.

*Structural medical review and physical examination.* From an open interview with both patient and caregiver, data are collected on presence of comorbidities (cardiovascular, cerebrovascular, endocrine, somatic, and psychiatric diseases), family history diagnoses of dementia and Parkinsonism, medication use and smoking behavior and alcohol consumption. In addition, a physical examination is conducted, which includes measurement of blood pressure, body mass index, gait disturbances, and extrapyramidal symptoms.

*Diagnosis.* At baseline, diagnoses are made by a multidisciplinary team, consisting of neuropsychiatrists, neuropsychologists, and research nurses, based on standardized clinical assessments, an extensive neuropsychological assessment, blood tests, MRI cerebrum, and for a subsample of patients CSF markers of AD pathology. A diagnosis of dementia is made according to DSM-IV criteria^11^ up to 2016, and from 2017 onwards DSM-5 criteria for major neurocognitive disorder are used.^12^ Etiological dementia diagnoses are made according to standardized clinical criteria for AD (NINCDS-ADRDA criteria, core clinical criteria,^3,13^ vascular dementia (VaD, NINDS-AIREN criteria),^14^ Lewy body dementia,^15^ and frontotemporal dementia (FTD).^16^ A diagnosis of MCI is based on the core clinical National Institute on Aging-Alzheimer's Association (NIA-AA) criteria, which entails that patients have objectified impaired cognitive functioning, but sufficiently preserved functional performance such that a dementia diagnosis cannot be made.^17^ All other patients are classified as SCD, indicating the presence of a cognitive complaint initiating the referral to the memory clinic, but the absence of objectified cognitive impairments or impairment in daily functioning. The broad range of variables included allow a retrospective application of newly designed diagnostic and research criteria based on biomarkers. At follow-up, diagnoses are based on a standardized history taking, to investigate the current situation and change since the last assessment, plus outcome measures concerning cognitive and daily functioning, and neuropsychiatric symptoms (elaborated upon below).

#### Clinical measures of daily functioning

The CDR, a clinician-rated global rating of dementia severity, is obtained via a semi-structured interview with patient and caregiver.^9^ The severity of impairment on basic and instrumental activities in daily life is evaluated with the Disability Assessment for Dementia (DAD), based on an interview with the caregiver.^18^ Self-reported change in cognitive functioning is assessed with the 4-item questionnaire on Subjective Cognitive Functioning (SCF).

#### Neuropsychological assessment

The neuropsychological assessment consists of a standardized battery that incorporates the harmonized test battery of the Dutch Parelsnoer Initiative, pearl neurodegenerative diseases^19^ and the more recent Dutch “Monodisciplinary guideline for neuropsychological assessment in MCI and dementia”.^20^ The BBACL battery includes: the standardized MMSE to assess global cognition^21^; the word-list of the Groninger Intelligence Test-2 (GIT-2)^22^ for an indication of premorbid IQ, the 15-Word Verbal Learning Test (VLT)^23,24^ and the stories from the Rivermead Behavioral Memory Test (RBMT)^25^ to assess episodic memory; the Visual Association Test (VAT) to assess implicit associative visual learning^26^; the digit-span (forward, backward) of the WAIS III or WAIS IV (additionally, sorting) to assess verbal span and working memory^27,28^; the fluency task (1-min animals and professions) to assess semantic verbal word fluency^29^; the Letter Digit Substitution Test (LDST),^30^ Stroop Color Word Test (SCWT)^31^ (card 1) and Concept Shifting Test (CST)^32^ (part 1) or Trail Making Test (TMT) part A^33^ to assess information processing speed; the SCWT (card 2 and 3) and CST (part C) or TMT B to assess attention and executive functioning. Cognitive performances are converted to z-scores based on age, sex and education adjusted test specific normative data for the Dutch population.^24,29–32^

#### Neuropsychiatric symptoms

Presence, frequency, severity, and caregiver burden of twelve neuropsychiatric symptoms (NPS) is evaluated using the informant-based Neuropsychiatric Inventory (NPI).^34^ Domains that are assessed include delusions, hallucinations, agitation, depression, anxiety, apathy, irritability, euphoria, disinhibition, aberrant motor behavior, night-time behavior disturbances, and appetite and eating abnormalities. By multiplying the frequency and severity ratings, a domain score is obtained. A domain score of one or more means that the symptom was present. The Geriatric Depression Scale-15 items (GDS-15)^35^ is used to screen for self-reported depressive symptoms.

#### Patient (health-related) quality of life and resource use

Quality of life is assessed with a self-report measure, the EuroQoL-5D3L.^36^ In a subpopulation, wellbeing in terms of capabilities is proxy rated by the caregiver with the ICECAP-O (in terms of attachment, security, role, enjoyment and control), providing an index which can be used in the economic evaluation of interventions^37^; the Resource Utilization in Dementia (RUD) informal care^38^ and the Care-related Quality of Life (CarerQol).^39^

#### Structural brain imaging

At baseline, all patients who have no contraindication for MR-procedures have an MRI cerebrum conform the harmonized SOP of the PSI-NDZ.^19^ All MR-images are performed on the same 3.0 Tesla Philips scanner. Currently, a subset of the MR-images is being read and rated for research purposes according to the following visual rating scales: medial temporal lobe atrophy (MTA),^40^ global cortical atrophy (GCA),^41^ posterior atrophy ratings (Koedam rating),^42^ white matter hyperintensities (Fazekas score).^43^ Further, lacunar and cortical infarcts, and (sub)cortical microbleeds are counted.

#### Biomarker assessment

For research purposes, additional blood (for plasma, serum and DNA) and (in a subset) CSF and/or tears samples are collected. Processing and storage is done conform the harmonized SOP of the PSI-NDZ^19^ at the Biobank Maastricht, the ISO (9001-2015) certificated Biobank of the MUMC+. In general, biomarker measurements are done blinded for all clinical data.

#### Serum and plasma samples

At baseline, venous blood samples are collected (10 ml clotted blood for serum and 8 ml EDTA blood for plasma). After collection, samples are being centrifuged, aliquoted into 0.5 ml vials and stored at −80°C in the biobank.

#### DNA

For scientific purposes, DNA is extracted from the buffy coat of an EDTA whole blood sample. DNA is isolated by the department of Clinical Genetics of the MUMC+. After isolation, DNA is stored in four cups at −80°C. In a subset, Apolipoprotein E (*APOE*) genotype is determined on genomic DNA using the polymerase chain reaction (PCR) technique.^44^

#### Cerebrospinal fluid

In a subpopulation, CSF is collected via a lumbar puncture. For clinical purposes, AD specific biomarkers, including Amyloid-beta 1-42 (Aβ_42_), total-tau (t-tau), and phosphorylated tau (p-tau181) levels are assessed using the INNOTEST enzyme-linked immunosorbent assays (ELISA) kit (Fujirebio Diagnostics, Belgium) at the department of Neurochemistry, Radboud University MC, Nijmegen, the Netherlands. For this assay, regularly clinical cut off scores for normal levels are: Aβ_42_ > 500 pg/ml, total-tau <350 pg/ml, and p-tau <85 pg/ml.

For research purposes, 5 ml additional CSF is collected and stored in polypropylene tubes. After collection, CSF is centrifuged at room temperature and aliquoted in 0.25 and 0.5 ml vials and stored at the biobank at −80°C until analysis.

#### Tear fluid

Tear fluid is collected from both eyes using Schirmer tear strips (TEAR strips, Contacare Ophthalmics and diagnostics, Gujarat, India). After collection, two tear strips are stored at the biobank.

#### Follow-up assessment

At one, two, five, and ten years after the baseline assessment, patients who had no dementia at baseline, are invited to participate in a follow-up assessment, where the same clinical and neuropsychological data is collected. See Supplemental Table 1 for a more detailed overview of which data were collected at each time point. In case participants are not able to visit the memory clinic, the patient will be visited at home. If not available for follow-up assessment at all, a standardized interview with participant or caregiver is conducted by telephone to obtain information on the reason for drop-out, present symptoms and the CDR score. Refusal at one follow-up moment does not exclude re-entry into the study at a later time point. Once the disease stage of dementia is reached, patients are not invited for further follow-up assessments.

#### Statistics

Statistical analyses were performed using SPSS Version 29 (IBM) for Mac OS X. Differences between diagnostic groups were analyzed using chi-square tests for categorical variables and ANOVAs for continuous variables. Non-parametric tests were used in case of a non-normal distribution. Cognitive test performances were transformed into age, sex and education corrected z-scores, based on Dutch normative data of healthy controls. Extreme z-scores were winsorized between a lower (−5) and upper (+5) boundary.

## Results

Table 1 presents the baseline characteristics of the first 920 patients, stratified by baseline diagnosis. These participants were on average 68.4 (±11.3) years old (range 31–93 years) and 41.6% were females. The mean MMSE score was 27.0 (±2.6) (range 20–30). At baseline, 364 (39.6%) patients had a diagnosis of SCD, 368 (40.0%) had a diagnosis of MCI and 188 (20.4%) patients had a clinical diagnosis of dementia, of whom 109 (58.0%) were diagnosed with probable/possible AD dementia, 23 (12.2%) with probable/possible VaD, 37 (19.7%) with mixed dementia (AD/VaD), 6 (3.2%) with FTD, 4 (2.1%) with Parkinson dementia, 1 (0.5%) with progressive (non-fluent) aphasia, 7 (3.7%) with dementia “other” and 1 (0.5%) as dementia not specified.

**Table 1.** Baseline demographic characteristics by clinical diagnosis of participants in the BBACL cohort.

	SCD (N = 364)	MCI (N = 368)	Dementia (N = 188)	Total (N = 920)	p
*Demographics*					
Age	62.2 (11.1)	71.0 (9.8)	75.4 (7.6)	68.4 (11.3)	<0.001^3^
Female, N (%)	136 (37.4)	157 (42.7)	90 (47.9)	383 (41.6)	0.052^2^
Education, N (%)					0.015^2^
Low	114 (31.3)	132 (35.9)	87 (46.3)	333 (36.2)	
Middle	150 (41.2)	146 (39.7)	64 (34.0)	360 (39.1)	
High	100 (27.5)	90 (24.5)	37 (19.7)	227 (24.7)	
*Comorbidities, N (%)* ^7^					
Cardiovascular	178 (49.0)	211 (57.5)	107 (57.2)	496 (54.1)	0.013^2^
Cerebrovascular	35 (9.6)	56 (15.5)	24 (12.9)	115 (12.6)	0.035^2^
Endocrine	53 (14.6)	85 (23.4)	49 (26.5)	187 (20.5)	0.001^2^
Psychiatric	135 (37.1)	84 (23.2)	36 (19.4)	255 (28.0)	<0.001^2^
Somatic	286 (78.6)	296 (81.1)	152 (81.3)	734 (80.1)	0.579^2^
*Cognition*					
MMSE	28.7 (1.4)	26.9 (2.3)	24.2 (2.5)	27.0 (2.6)	<0.001^1^
VLT immediate^4^	0.2 (1.1)	−1.2 (1.2)	−2.0 (1.1)	−0.8 (1.4)	<0.001^3^
VLT delayed^4^	0.1 (1.1)	−1.5 (1.3)	−2.3 (1.0)	−1.0 (1.5)	<0.001^3^
VLT recognition^4^	−0.0 (0.9)	−0.9 (1.3)	−1.5 (1.3)	−0.7 (1.3)	<0.001^3^
CST A^4^	−0.3 (1.2)	−0.8 (1.3)	−1.2 (1.7)	−0.7 (1.4)	<0.001^3^
CST B^4^	−0.3 (1.3)	−1.2 (1.6)	−1.6 (1.9)	−0.9 (1.6)	<0.001^3^
CST C^4^	−0.2 (1.1)	−1.1 (1.6)	−1.8 (1.8)	−0.8 (1.6)	<0.001^3^
Animal fluency^4^	−0.2 (0.9)	−1.1 (0.9)	−1.6 (0.8)	−0.8 (1.0)	<0.001^3^
*Scales and questionnaires*					
CDR, N (%)^7^					
0	48 (13.2)	14 (3.8)	3 (1.6)	65 (7.1)	<0.001^2^
0.5	308 (84.6)	333 (90.5)	130 (69.1)	771 (83.8)	
1.0	8 (2.2)	21 (5.7)	55 (29.3)	84 (9.1)	
DAD	92.7 (10.5)	87.3 (11.9)	75.0 (17.5)	86.9 (14.3)	<0.001^1^
EuroQoL-5D-VAS	69.1 (14.3)	71.0 (14.9)	72.3 (15.7)	70.5 (14.8)	0.017^1^
NPI-total	12.6 (13.4)	12.6 (11.7)	18.1 (15.4)	13.7 (13.4)	<0.001^1^
GDS-15	3.9 (3.0)	3.3 (2.8)	2.8 (2.3)	3.4 (2.8)	<0.001^1^
SCF	−3.7 (2.7)	−2.9 (2.6)	−2.3 (2.3)	−3.1 (2.6)	<0.001^3^
*Biomarkers*					
*APOE* ε4 carrier, N (%)^7^	28 (45.2)	20 (38.5)	17 (63.0)	65 (46.1)	0.115^2^
CSF present, N (%)^6, 7^	83 (22.8)	63 (17.1)	30 (16.0)	176 (19.1)	0.101^2^
CSF Aβ_42_, pg/ml, % abnormal^7^	1066.9 (332.1)	914.1 (424.5)	715.7 (243.3)	951.0 (377.0)	<0.001^1^
CSF t-tau, pg/ml^7^	246.3 (161.6)	414.6 (264.6)	691.2 (386.1)	383.9 (296.0)	<0.001^1^
CSF p-tau, pg/ml^7^	50.0 (27.5)	67.2 (33.5)	95.4 (36.5)	64.1 (35.2)	<0.001^1^
*Other parameters*					
BMI (kg/m2)	26.9 (5.1)	26.6 (4.5)	25.9 (4.2)	26.6 (4.7)	0.096^3^

^1^ Kruskal-Wallis rank sum test, ^2^Pearson's Chi-squared test, ^3^ANOVA, ^4^z-scores based on deviations from the norm based on age and education, ^5^measured with VAS-scale, ^6^clinical diagnostic measurements, ^7^Note that % are frequencies of people having data available. SCD: subjective cognitive decline; MCI: mild cognitive impairment; BMI; body mass index; MMSE: Mini-Mental State Evaluation; DAD: Disability Assessment for Dementia; CDR: Clinical Dementia Rating Scale; VLT: 15 word-Verbal Learning Test; CST: Concept Shifting Task; NPI: Neuropsychiatric Inventory; GDS-15: Geriatric Depression Scale-15 items; *APOE*: Apolipoprotein E; CSF: cerebrospinal fluid; Aβ: amyloid-beta; t-tau: total tau; p-tau: phosphorylated tau.

As expected, people with dementia were significantly older than people with MCI or SCD (Table 1). The groups differed significantly on all cognitive tests. Post-hoc analyses showed that all cognitive test performances were significantly worse in people with dementia than in people with MCI or SCD, and in those with MCI significantly worse than in those with SCD, except no difference was found on the CST B between people with MCI and dementia (p = 0.093).

The prevalence of comorbidities related to kidney, lung, and liver diseases was comparable across diagnostic groups. Significant differences were found for cardiovascular, cerebrovascular, endocrine, and psychiatric comorbidities (Table 1). Independent of syndrome diagnosis, the prevalence of any neuropsychiatric symptom was relatively high: 84.9% of the patients scored positive (domain score > 0) on at least one of the twelve NPI domains. The proportion of patients with at least one NPS was greatest in the dementia subgroup (91.9%, versus 84.1% in MCI and 82.0% in SCD, χ^2^ = 9.52, p = 0.009). The most prevalent NPS were irritability (56.4%), depression (46.1%), apathy (36.2%), sleep problems (33.9%), and anxiety (27.5%). The mean score on the GDS-15 was 3.4 points. Diagnostic groups differed from each other, with higher GDS-15 scores in SCD than in MCI or dementia, but no difference between MCI and dementia groups. Also the mean SCF scores differed between groups. Post hoc analyses showed that people with SCD rated their cognitive functioning lower compared to those with MCI or dementia, but MCI differed not differ from dementia. Diagnostic groups also differed in DAD scores. Post hoc analyses showed differences between all groups (dementia < MCI < SCD).

For 141 patients, *APOE* genotype has been determined in the context of the PSI-NDZ substudy,^19^ of whom 65 (46.1%) had one or two ε4 alleles (specifically: ε4ε4 n = 11 (7.8%), ε3ε4 n = 50 (35.5%), and ε2ε4 n = 4 (2.8%), while 76 patients (53.9%) did not carry an ε4 allele (ε3ε3 n = 63 (44.7%), ε2ε3 n = 11 (7.8%), and ε2ε2 n = 2 (1.4%)). CSF measures for diagnostic purposes were available for 176 (19.1%) patients. Diagnostic groups differed significantly from each other in terms of Aβ_42_, t-tau and p-tau levels (Table 1). Post hoc analyses showed that CSF Aβ_42_ concentrations in SCD were more normal compared to those in MCI or dementia, but MCI did not differ from dementia (p = 0.076), and CSF t-tau and p-tau concentrations were significantly different between all groups (dementia > MCI > SCD).

## Discussion

BBACL is an ongoing prospective clinical cohort study, collecting a wide range of outcomes for research on natural disease course and determinants of progression in a memory clinic setting. As part of the multifactorial approach, BBACL includes outcomes on biomarkers, comorbidities, cognition, NPS, daily functioning, and health-economic measures, with currently 920 consecutively included participants.

In terms of cohort characteristics, 20.4% of the people were diagnosed with dementia at study entry. The other part was referred with milder cognitive impairments. This is in line with findings from a survey among 92 memory clinics in the Netherlands, which reported that the proportion of memory clinic visitors without dementia has increased substantially over the last decades.^45^ One of the reasons could be that people are seeking for help in a memory clinic at an earlier stage, which might reflect the increased recognition of the public and health-care providers of the early signs and symptoms of dementia.^46^ Early referrals might also create a window of opportunity wherein timely interventions and individualized care can be provided.^8^ The majority of persons with dementia had AD-type dementia, followed by mixed AD/VaD and then VaD, and only a limited number of other types of dementia (e.g., FTD or Lewy body dementia) were included, which reflects clinical practice.

As expected, cognitive performances and scores on clinical rating scales differed clearly between the diagnostic groups. The extent of cognitive dysfunctioning in the dementia group was relatively mild compared to other dementia cohorts, which can be explained by the inclusion of people with mild dementia only. This has consequences for the generalizability of results to the whole dementia spectrum, as moderate to severe stages of dementia are not included in the study.

The majority of the people reported one or more NPS. Most prevalent were irritability, depression, apathy, sleep problems, and anxiety. Prevalence rates of NPS were comparable to other cohorts in the field.^19,47^ In BBACL, the prevalence of NPS increased across the diagnostic groups (SCD to MCI to dementia). Self-reported depressive symptoms are slightly higher than those reported elsewhere.^48^ People with SCD have significantly more often a (history of) psychiatric disorders compared to MCI and dementia. This may be related to the fact that the memory clinic of the MUMC + is embedded in the psychiatry department.

In a subgroup of patients of whom *APOE* status was available, we observe that *APOE* ε4, a well-known genetic risk factor for AD, was present in 45.2% of the SCD group. This is in line with findings of Jessen et al. (2018) and indicates enrichment of *APOE* ε4 in the SCD group compared to the general Dutch population (frequency of 16.3%),^48,49^ and implies an increased risk for developing AD dementia in all non-demented memory clinic visitors. As expected, group differences were found for the CSF biomarker levels of Aβ_42_, t-tau and, p-tau.

Strengths of the BBACL cohort include the inclusion of people across the broad AD spectrum and multimodal data well integrated in regular patient care. The MUMC + is an academic hospital, but because it is geographically the only hospital in the area, it also functions as a regional hospital. The BBACL cohort is therefore representative of a general memory clinic. The study includes a wide variety of clinical variables over time and utilizes a standardized and validated neuropsychological test battery. This battery is in line with the nationally standardized PSI-NDZ battery. The large overlap with SOPs of the Dutch PSI-NDZ^19^ provides unique opportunities for future combination of other cohort data. BBACL allows for the investigation of the causes and natural course of cognitive functioning and other long-term outcomes. The clinical, cognitive, neuropsychiatric, brain imaging, blood, genetic and CSF markers included allow for the design of individualized prediction models. In addition, the extensive biobanking allows for the investigation of well-established and newly identified biomarkers for cognitive disorders. The deeply phenotyped data in combination with the biobanking possibilities of BBACL were already part of several collaborations in the past as apparent from multiple publications.^50–54^ Future availability of AD blood biomarkers and (automatically derived) volumetric measurements of MRI subfields will further strength the etiological underpinnings of cognitive impairments in this cohort. Although this is a local cohort, the use of comprehensive metadata and detailed data dictionaries, together with harmonized cognitive measures, provides a strong foundation for comparison and harmonization with international cohorts. Building on these resources, future collaboration with international biobank networks and AD consortia could facilitate cross-cohort analyses, replication, and external validation of findings, thereby enhancing the broader applicability and research potential of the cohort. Another strength is the recent inclusion of health-economic outcomes, which are scarce in pre-dementia studies. For one, the current limits on the effectiveness of interventions to fully prevent dementia make maintaining and optimizing QoL one of the most pivotal goals of dementia management. The BBACL data allows to determine the health-economic impact of (in)formal dementia care. Further, the utility of diagnostic tests in terms of costs, next to their impact on diagnostic confidence or diagnostic decision making, can be determined.^55^

Despite these strengths, including the opportunity to follow participants for up to 10 years, several limitations inherent to a longitudinal, memory clinic-based observational cohort should be acknowledged. Clinical recruitment may have introduced selection and referral bias, potentially limiting the generalizability of our findings to findings from the general population. The extended follow-up period also creates the possibility of attrition and selective survival, as participants who remain in follow-up may differ systematically from those lost to follow-up. Future longitudinal statistical analyses will account for patterns of missing data and attrition and assess their potential impact through appropriate statistical methods and sensitivity analyses. Furthermore, given the observational nature of the study, causal relationships cannot be established, although the longitudinal design allows for a clearer assessment of temporal associations. Finally, as biomarkers were primarily assessed at baseline, we could not fully capture how longitudinal changes in biomarkers relate to cognitive trajectories over time.

### Conclusion

BBACL is an ongoing prospective cohort study of a memory clinic in the Netherlands with follow-up data up to ten years to be available in future. It includes a wide variety of multimodal data, which provides unique opportunities to model trajectories of disease progression in relation to a diverse set of clinical, biological, and health-economic outcomes. Baseline biomarkers and longitudinal outcomes of cognition and NPS are the key outcome domains and their longitudinal changes and dynamic relations will be further explored.

## Supplemental Material

sj-docx-1-alr-10.1177_25424823261496546 - Supplemental material for Biobank Alzheimer Center Limburg study: A multimodal longitudinal dementia biobank Supplemental material, sj-docx-1-alr-10.1177_25424823261496546 for Biobank Alzheimer Center Limburg study: A multimodal longitudinal dementia biobank by Inez H.G.B. Ramakers, Leonie C.P. Banning, Willemijn J. Jansen, Lieke Bakker, Ron L.H. Handels, Sebastian Köhler, Pieter Jelle Visser, Stephanie J.B. Vos, Pauline Aalten and Frans R.J. Verhey in Journal of Alzheimer's Disease Reports

## Data Availability

The datasets used during the current study are available from the corresponding author on reasonable request. External collaborations are welcomed by the BBACL research group. Researchers with specific research questions are encouraged to contact the study coordinators (Dr Inez Ramakers, email: i.ramakers@maaastrichtuniversity.nl or Prof. Dr Frans Verhey, email: f.verhey@maastrichtuniversity.nl). Data requests will be evaluated in the BBACL scientific board.

## References

[1] World Health Organization. Global status report on the public health response to dementia. World Health Organization, 2021.

[2] Wimo A Jonsson L Bond J , et al. The worldwide economic impact of dementia 2010. Alzheimers Dement 2013; 9: 1–11 e13. 10.1016/j.jalz.2012.11.006 23305821

[3] McKhann GM Knopman DS Chertkow H , et al. The diagnosis of dementia due to Alzheimer’s disease: recommendations from the national institute on aging-Alzheimer’s association workgroups on diagnostic guidelines for Alzheimer’s disease. Alzheimers Dement 2011; 7: 263–269. 10.1016/j.jalz.2011.03.005 PMC3312024 21514250

[4] Jansen WJ Ossenkoppele R Knol DL , et al. Prevalence of cerebral amyloid pathology in persons without dementia: a meta-analysis. JAMA 2015; 313: 1924–1938. 10.1001/jama.2015.4668 PMC4486209 25988462

[5] Petersen RC. Mild cognitive impairment as a diagnostic entity. J Intern Med 2004; 256: 183–194. 10.1111/j.1365-2796.2004.01388.x 15324362

[6] Jessen F Amariglio RE van Boxtel M , et al. A conceptual framework for research on subjective cognitive decline in preclinical Alzheimer’s disease. Alzheimers Dement 2014; 10: 844–852. 10.1016/j.jalz.2014.01.001 PMC4317324 24798886

[7] Vos SJ van Rossum IA Verhey F , et al. Prediction of Alzheimer disease in subjects with amnestic and nonamnestic MCI. Neurology 2013; 80: 1124–1132. 10.1212/WNL.0b013e318288690c 23446677

[8] de Vugt ME Verhey FR . The impact of early dementia diagnosis and intervention on informal caregivers. Prog Neurobiol 2013; 110: 54–62. 10.1016/j.pneurobio.2013.04.005 23689068

[9] Morris JC. The clinical dementia rating (CDR): current version and scoring rules. Neurology 1993; 43: 2412–2414. 10.1212/wnl.43.11.2412-a 8232972

[10] Folstein MF Folstein SE McHugh PR . Mini-mental state”. A practical method for grading the cognitive state of patients for the clinician. J Psychiatr Res 1975; 12: 189–198. 10.1016/0022-3956(75)90026-6 1202204

[11] American Psychological Association. Diagnostic and statistical manual of mental disorders (4th edition). American Psychological Association, 1994.

[12] American Psychological Association. Diagnostic and statistical manual of mental disorders (5th edition). American Psychological Association, 2022.

[13] McKhann G Drachman D Folstein M , et al. Clinical diagnosis of Alzheimer’s disease: report of the NINCDS-ADRDA work group under the auspices of department of health and human services task force on Alzheimer’s disease. Neurology 1984; 34: 939–944. 10.1212/wnl.34.7.939 6610841

[14] Roman GC Tatemichi TK Erkinjuntti T , et al. Vascular dementia: diagnostic criteria for research studies. Report of the NINDS-AIREN international workshop. Neurology 1993; 43: 250–260. 10.1212/wnl.43.2.250 8094895

[15] McKeith IG Galasko D Kosaka K , et al. Consensus guidelines for the clinical and pathologic diagnosis of dementia with Lewy bodies (DLB): report of the consortium on DLB international workshop. Neurology 1996; 47: 1113–1124. 10.1212/wnl.47.5.1113 8909416

[16] Neary D Snowden JS Gustafson L , et al. Frontotemporal lobar degeneration: a consensus on clinical diagnostic criteria. Neurology 1998; 51: 1546–1554. 10.1212/wnl.51.6.1546 9855500

[17] Albert MS DeKosky ST Dickson D , et al. The diagnosis of mild cognitive impairment due to Alzheimer’s disease: recommendations from the national institute on aging-Alzheimer’s association workgroups on diagnostic guidelines for Alzheimer’s disease. Alzheimers Dement 2011; 7: 270–279. 10.1016/j.jalz.2011.03.008 PMC3312027 21514249

[18] Gelinas I Gauthier L McIntyre M , et al. Development of a functional measure for persons with Alzheimer’s disease: the disability assessment for dementia. Am J Occup Ther 1999; 53: 471–481. 10.5014/ajot.53.5.471 10500855

[19] Aalten P Ramakers IH Biessels GJ , et al. The Dutch Parelsnoer institute–neurodegenerative diseases; methods, design and baseline results. BMC Neurol 2014; 14: 254. 10.1186/s12883-014-0254-4 PMC4301568 25551191

[20] Ramakers I Gruters A Stiekema A , et al. Neuropsychologisch onderzoek bij de diagnostiek van lichte cognitieve stoornissen (MCI) en dementie: de weg naar een monodisciplinaire richtlijn. Tijdschr. Neuropsychol 2020; 15: 91–105.

[21] Kok R Verhey F . Gestandaardiseerde versie van de MMSE: een toelichting bij de scoring. Utrecht 2002.

[22] Luteijn F Barelds D . Herziening van de GIT. Handleiding bij de GIT-2. 2005.

[23] Brand N Jolles J . Learning and retrieval rate of words presented auditorily and visually. J Gen Psychol 1985; 112: 201–210. 10.1080/00221309.1985.9711004 4056765

[24] Van der Elst W van Boxtel MP van Breukelen GJ , et al. Rey’s verbal learning test: normative data for 1855 healthy participants aged 24–81 years and the influence of age, sex, education, and mode of presentation. J Int Neuropsychol Soc 2005; 11: 290–302. 10.1017/S1355617705050344 15892905

[25] Van Balen H Groot Zwaaftink A . The Rivermead Behavioural Memory Test. Nederlandstalige bewerking. Handleiding. Swets & Zeitlinger, 1987.

[26] Lindeboom J Schmand B Tulner L , et al. Visual association test to detect early dementia of the Alzheimer type. J Neurol Neurosurg Psychiatry 2002; 73: 126–133. 10.1136/jnnp.73.2.126 PMC1737993 12122168

[27] Wechsler D. WAIS-IV-NL: Wechsler adult intelligence scale: Nederlandse bewerking. Pearson Assessment and information BV, 2012.

[28] Wechsler D. WAIS III, Nederlandstalige Bewerking: Afname en Scoringshandleiding. Swets Test Publishers, 2000.

[29] Van der Elst W Van Boxtel MP Van Breukelen GJ , et al. Normative data for the animal, profession and letter M naming verbal fluency tests for Dutch speaking participants and the effects of age, education, and sex. J Int Neuropsychol Soc 2006; 12: 80–89. 10.1017/S1355617706060115 16433947

[30] van der Elst W van Boxtel MP van Breukelen GJ , et al. The letter digit substitution test: normative data for 1,858 healthy participants aged 24–81 from the Maastricht aging study (MAAS): influence of age, education, and sex. J Clin Exp Neuropsychol 2006; 28: 998–1009. 10.1080/13803390591004428 16822738

[31] Van der Elst W Van Boxtel MP Van Breukelen GJ , et al. The Stroop color-word test: influence of age, sex, and education; and normative data for a large sample across the adult age range. Assessment 2006; 13: 62–79. 10.1177/1073191105283427 16443719

[32] Van der Elst W Van Boxtel MP Van Breukelen GJ , et al. The concept shifting test: adult normative data. Psychol Assess 2006; 18: 424–432. 10.1037/1040-3590.18.4.424 17154763

[33] Reitan RM. Validity of the trail making test as an indicator of organic brain damage. Percept Mot Skills 1958; 8: 271–276.

[34] Cummings JL Mega M Gray K , et al. The neuropsychiatric inventory: comprehensive assessment of psychopathology in dementia. Neurology 1994; 44: 2308–2314. 10.1212/wnl.44.12.2308 7991117

[35] Yesavage JA Sheikh JI . Geriatric depression scale (GDS) recent evidence and development of a shorter version. Clin Gerontol 1986; 5: 165–173.

[36] EuroQol G. Euroqol–a new facility for the measurement of health-related quality of life. Health Policy 1990; 16: 199–208. 10.1016/0168-8510(90)90421-9 10109801

[37] Coast J Flynn TN Natarajan L , et al. Valuing the ICECAP capability index for older people. Soc Sci Med 2008; 67: 874–882. 10.1016/j.socscimed.2008.05.015 18572295

[38] Wimo A Jonsson L Zbrozek A . The resource utilization in dementia (RUD) instrument is valid for assessing informal care time in community-living patients with dementia. J Nutr Health Aging 2010; 14: 685–690. 10.1007/s12603-010-0316-2 PMC12880235 20922346

[39] Brouwer WB van Exel NJ van Gorp B , et al. The CarerQol instrument: a new instrument to measure care-related quality of life of informal caregivers for use in economic evaluations. Qual Life Res 2006; 15: 1005–1021. 10.1007/s11136-005-5994-6 16900281

[40] Scheltens P Leys D Barkhof F , et al. Atrophy of medial temporal lobes on MRI in “probable” Alzheimer’s disease and normal ageing: diagnostic value and neuropsychological correlates. J Neurol Neurosurg Psychiatry 1992; 55: 967–972. 10.1136/jnnp.55.10.967 PMC1015202 1431963

[41] Pasquier F Leys D Weerts JG , et al. Inter- and intraobserver reproducibility of cerebral atrophy assessment on MRI scans with hemispheric infarcts. Eur Neurol 1996; 36: 268–272. 10.1159/000117270 8864706

[42] Lehmann M Koedam EL Barnes J , et al. Posterior cerebral atrophy in the absence of medial temporal lobe atrophy in pathologically-confirmed Alzheimer’s disease. Neurobiol Aging 2012; 33: 627 e621–627 e612. 10.1016/j.neurobiolaging.2011.04.003 PMC3657170 21596458

[43] Fazekas F Barkhof F Wahlund LO , et al. CT And MRI rating of white matter lesions. Cerebrovasc Dis 2002; 13: 31–36. 10.1159/000049147 11901240

[44] Bekers O op den Buijsch RA de Vries JE , et al. Capillary electrophoretic detection in apolipoprotein E genotyping. Electrophoresis 2002; 23: 1878–1881. 10.1002/1522-2683(200206)23:12<1878::AID-ELPS1878>3.0.CO;2-G 12116131

[45] Gruters AAA Ramakers I Kessels RPC , et al. Development of memory clinics in The Netherlands over the last 20 years. Int J Geriatr Psychiatry 2019; 34: 1267–1274. 10.1002/gps.5132 PMC6767517 31034652

[46] Peters ME Lyketsos CG . Beyond memory: a focus on the other neuropsychiatric symptoms of dementia. Am J Geriatr Psychiatry 2015; 23: 115–118. 10.1016/j.jagp.2014.11.002 PMC4293074 25575934

[47] Banning LCP Ramakers I Kohler S , et al. The association between biomarkers and neuropsychiatric symptoms across the Alzheimer’s disease spectrum. Am J Geriatr Psychiatry 2020; 28: 735–744. 10.1016/j.jagp.2020.01.012 32088096

[48] Jessen F Spottke A Boecker H , et al. Design and first baseline data of the DZNE multicenter observational study on predementia Alzheimer’s disease (DELCODE). Alzheimers Res Ther 2018; 10: 15. 10.1186/s13195-017-0314-2 PMC5802096 29415768

[49] Corbo RM Scacchi R . Apolipoprotein E (APOE) allele distribution in the world. Is APOE*4 a ‘thrifty’ allele? Ann Hum Genet 1999; 63: 301–310. 10.1046/j.1469-1809.1999.6340301.x 10738542

[50] van Gils V Vos SJ Jansen WJ , et al. The association between diabetes and Alzheimer’s disease pathophysiology. Alzheimers Dement 2021; 17: e050181.

[51] Kučikienė D Costa AS Banning LC , et al. The role of vascular risk factors in biomarker-based AT (N) groups: a German-Dutch memory clinic study. J Alzheimers Dis 2022; 87: 185–195. 10.3233/JAD-215391 35275532

[52] Delvenne A Gobom J Tijms B , et al. Cerebrospinal fluid proteomic profiling of individuals with mild cognitive impairment and suspected non-Alzheimer’s disease pathophysiology. Alzheimers Dement 2023; 19: 807–820. 10.1002/alz.12713 35698882

[53] Bakker L Köhler S Eussen SJ , et al. Correlations between kynurenines in plasma and CSF, and their relation to markers of Alzheimer’s disease pathology. Brain Behav Immun 2023; 111: 312–319. 10.1016/j.bbi.2023.04.015 37149106

[54] Gijs M Ramakers IH Visser PJ , et al. Association of tear fluid amyloid and tau levels with disease severity and neurodegeneration. Sci Rep 2021; 11: 22675. 10.1038/s41598-021-01993-x PMC8608861 34811435

[55] Handels RLH Wimo A Dodel R , et al. Cost-utility of using Alzheimer’s disease biomarkers in cerebrospinal fluid to predict progression from mild cognitive impairment to dementia. J Alzheimers Dis 2017; 60: 1477–1487. 10.3233/JAD-170324 29081416

