# Supplementary material for: Biobank Alzheimer Center Limburg study: A multimodal longitudinal dementia biobank
Source: J Alzheimers Dis Rep. 2026 Oct 8;10:25424823261496546. doi: 10.1177/25424823261496546 (PMC13662353; doi:10.1177/25424823261496546)
Supplement: Supplementary file 1 [file sj-docx-1-alr-10.1177_25424823261496546.docx]

**Supplemental Material**

**Biobank Alzheimer Center Limburg study: A multimodal longitudinal dementia biobank**

**Supplemental Table 1.** Standardized dataset BBACL cohort

| Content | |  | Baseline | Follow-up | |  |
| --- | --- | --- | --- | --- | --- | --- |
| *Patient-information* | | |  |  | |  |
| *Informed consent* | | | • | • | |  |
| *Demographics* | | |  |  | |  |
|  | | Age, gender, ethnicity, years of education, living situation | • | • | |  |
| *Structural medical review and physical examination* | | | | |  | |
|  | | Comorbidities, family history, medication use, smoking behavior, alcohol consumption, blood pressure, body mass index | • | • | |  |
|  | | Gait disturbances and extrapyramidal symptoms | • |  | |  |
| *Clinical information* | | |  |  | |  |
|  | | Diagnosis | • | • | |  |
| *Rating scales and questionnaires* | | |  |  | |  |
|  | *Clinical outcomes* | |  |  | |  |
|  | | CDR, SCF, DAD | • | • | |  |
|  | *HR-QoL and resource use* | |  |  | |  |
|  | | EuroQoL-5D-3L | • | • | |  |
|  | | ICECAP-O, CarerQoL, RUD informal care (subgroup) | • |  | |  |
|  | *Neuropsychiatric symptoms* | |  |  | |  |
|  | | GDS-15, NPI | • | • | |  |
| *Cognitive screening* | | |  |  | |  |
|  | | MMSE | • | • | |  |
| *Neuropsychological* tests | | |  |  | |  |
|  | | 15-VLT, RMBT stories, CST/TMT, SCWT, LDST, Digit Span, Fluency (1-minute animals and professions), VAT, GIT-word list | • | • | |  |
| *Imaging* | |  |  |  | |  |
|  | | MR cerebrum: T1, T2, Flair, DWI, DTI (optional), fMRI (optional)  MTA, GCA, Koedam, WMH, lacunar and cortical infarcts, (sub)cortical microbleeds | • |  | |  |
| *Biobank* | | |  |  | |  |
|  | | Blood (serum, plasma, DNA) | • |  | |  |
|  | | CSF (subset, optional) | • |  | |  |
|  | | Tears (subset, optional) | • |  | |  |

15-VLT: 15 item Verbal Learning Task; Carer-QoL: Care-related Quality of Life; CDR: Clinical dementia rating scale; CSF: cerebrospinal fluid markers; CST: Concept Shifting Task; DAD: Disability Assessment for Dementia; Euro-QoL-5D: European-Quality of Life- 5 Dimensions; GCA: Global Cortical Atrophy; GDS-15: Geriatric Depression Scale (15-items); GIT: Groninger Intelligentie Test; ICECAP-O: Investigating Choice Experiments for the Preferences of Older People, CAPability measure for Older people; LDST: Letter Digit Substitution Test; MMSE: Mini-Mental State Examination; MRI: magnetic resonance imaging; MTA: medial temporal lobe atrophy; NPI: Neuropsychiatric Inventory; RBMT: Rivermead Behavioural Memory Test; RUD: Resource Utilization in Dementia; SCWT: Stroop Color Word Test; TMT: Trail Making Test; VAT: Visual Association Test; WMH: white matter hyperintensities
